# Familial adenomatous polyposis of the colon

**DOI:** 10.1186/1897-4287-11-15

**Published:** 2013-10-22

**Authors:** Andrzej Plawski, Tomasz Banasiewicz, Pawel Borun, Lukasz Kubaszewski, Piotr Krokowicz, Marzena Skrzypczak-Zielinska, Jan Lubinski

**Affiliations:** 1 Institute of Human Genetics, Poznan, Poland; 2 University of Medical Sciences, Poznan, Poland; 3 Pomeranian Medical University, Szczecin, Poland

**Keywords:** FAP, *APC* gene, *MUTYH* gene, Colorectal cancer, Molecular diagnostics

## Abstract

Familial adenomatous polyposis (FAP) is a well-defined autosomal dominant predisposition to the development of polyposis in the colon and rectum at unusually early ages. The first symptoms of FAP are diarrhea and blood in the stool. Weight loss and weaknesses occur after the development of advanced tumour. The incidence of the FAP disorder is one per 10000 newborns. There are high levels of heterogeneity with regard to the number and timing of the occurrence of polyps. The classical form of FAP is characterized by the presence of more than 100 polyps, which appear in the second decade of life. The average time of occurrence of polyps is 15 years. The earliest symptoms of polyposis have been observed in a three-year-old child. The polyps are characterized by large potential for the development towards malignant tumour. Malignancy can occur from late childhood onwards. Attenuated adenomatous polyposis coli is characterized by a more benign course of disease in contrast to classical FAP. The occurrence of FAP is associated with mutations in the *APC* tumour suppressor gene, which was described in 1991. The *APC* gene is located on chromosome 5q21 and is involved in cell proliferation control. A recessive form of adenomatous polyposis is caused by mutations in the base excision repair gene - *MUTYH* gene. The *MUTYH* gene is involved in repairing DNA lesions as a result of oxidative DNA damage. MUTYH associated polyposis (MAP) is a predisposition to the development of polyps of the colon but the number of polyps is lower in comparison to classical FAP. The high risks of cancer observed in these two diseases make them important medical issues. Molecular studies of colonic polyposis have been performed in Poland for over fifteen years. A DNA Bank for Polish FAP patients was established at the Institute of Human Genetics in Poznan in which DNA samples from 600 FAP families have been collected.

## Introduction

Familial adenomatous polyposis (FAP) accounts for approximately 1% of all large intestinal tumour diagnoses [1]. The disease occurs *de novo* with the frequency of somewhere between 1 in 8000 to 1 in 10000 births and the age of disease penetrance varies quite considerably even between siblings [2]. However, the occurrence of a large intestine tumour at unusually young ages should provide a signal to perform a family anamnesis, which should identify whether this is a hereditary predisposition or not [3]. The occurrence of a single case of the disease does not rule out a high hereditary predisposition because a given patient may be the first carrier of the mutation. FAP symptoms occur earlier than in the most common hereditary predisposition to colorectal cancer, Lynch syndrome and generally start to appear in the second decade of life. Patients have, however been reported to be as young as 5 years of age and in the group of our youngest patients, one was only 3 years old when polyps were diagnosed [4]. The genetic basis of FAP is the presence of germline mutations in the *APC* gene in the case of FAP and in *MUTYH* gene – in the case of the recessive form of adenomatous polyposis, known as MUTYH adenomatous polyposis (MAP) [5].

The *APC* gene is a tumour suppressor gene that is involved in the control of β-catenin. Loss of APC function results in the constitutive activation of β-catenin, which culminates in cellular proliferation [6]. When cellular proliferation is allowed to occur unimpeded the probability of mutations occurring increase as the normal checkpoint controls are over-ridden. This leads to the accumulation of changes in genetic material and selection of malignant cells that are capable of escaping from their normal cellular constraints.

In the case of *APC* driven tumourigenesis, normally both alleles need to be lost prior to disease development. In patients with FAP they have inherited only one intact allele (i.e. functional copy of *APC*), which alters the probability of disease development [7].

### History of investigations of the *APC* gene

FAP was recognised as a heritable pathogenic syndrome already in the 1920s. In 1972, Gardener syndrome was described, which is a form of FAP characterised not only by the presence of hundreds or even thousands of adenomatous polyps in the intestine but also of osteomas and retinal hypertrophy (*congenital hypertrophy of the retinal pigment epithelium* - CHRPE). The occurrence of FAP was associated with the q21-q22 region of chromosome 5 on the basis of a large deletion discovered by cytogenetic analysis as well as research results from RFLP marker analysis in a patient with Gardener’s syndrome with advanced polyp growth in the colon [8]. Towards the end of the 1980s, association studies revealed a region on the long arm of chromosome 5, which encompassed *APC* and *MCC* genes separated from each other by 150 kbp. In 1991, the following three genes: *DP1*, *SRP19* and *DP2.5* found in the critical region were investigated in FAP patients. In the case of four unrelated FAP patients, four mutations were observed in the *DP2.5* (now known as *APC*), resulting in premature stop codons from which one was transmitted from generation to generation [1]. In the following year 79 FAP patients were examined and mutations in *APC* were observed in 67% of them. In a second study 92% of FAP cases, they were found to harbour mutations leading resulting in premature truncation mutations of *APC*[9]. In many countries, investigations were undertaken to study the occurrence of mutations in *APC* and a database of inherited and somatic mutations was established in which data from 826 inherited and 650 somatic mutations were collected [10]. Once *APC* had been identified, its function was investigated and found to bind with β-catenin which suggested its participation in cell adhesion [10].

In Poland, a DNA Bank of FAP patient samples was established in 1997 at the Institute of Human Genetics (IHG) of the Polish Academy of Sciences (PAS) in Poznan in cooperation with Polish Polyposis Registry. Over 1000 DNA samples have been collected in the IHG PAS in Poznan within the framework of investigations into genetic susceptibilities for the development of neoplastic diseases associated with the presence of polyps in the gastrointestinal tract. Currently, in the DNA Bank there are over 1000 DNA samples stored from 700 FAP patients derived from 600 families. Moreover, material from risk groups in which no symptoms of the disease were observed at registration has also been gathered. Molecular studies have resulted in the identification of mutations in *APC* in 294 families [11-16].

### *APC* structure and APC protein function

*APC* (*adenomatous polyposis coli*) is located on chromosome 5 in the q21 region and contains 21 exons [17]. A characteristic attribute of *APC* is the occurrence of a large exon 15, which encompasses over 70% of the coding sequence. The expression of the gene is observed in all tissues and the mRNA transcript is 8538 nucleotides long [1,10]. The first exons can form tissue-specific alternative transcripts [18], for example an *APC* brain specific gene transcript for which the start codon is located in the BS (*brain specific*) exon. Elimination of codon 1, which encodes the super helix domain, known to be required for homo- or heterodimerisation, affects protein product functionality not [17]. Alternative splicing of exon 9 has been associated with the occurrence of the *attenuated adenomatous polyposis coli* (AAPC) [19]. A similar effect was recorded in one of two families with mutations at the 3’ end of exon 9 where, in one case, a modifying effect was observed leading to a milder FAP phenotype. That evidences suggests that the location of mutation as well as the effect of alternative splicing exerts an influence on the disease expression.

Another very interesting phenomenon is the differential splicing out of exons 14 and 15 of *APC* (which comprises nearly 70% of the gene), with the remaining fragment binding with the 3’ end of the *SRPI* gene. Excising exon 14 or 15 results in the development of two isoforms differing from each other by their ability to bind microtubules and β-catenin as well as by the 3’ region which does not undergo translation and can affect mRNA stability and protein translation [20,21]. An alternative gene assembly, in this case, is associated with the regulation of the APC protein activity and appears to suggest that it fulfils a number of different functions in the cell, especially, in the alternative assembly of the protein where over 75% of exons take part.

The full-length APC protein contains 2843 amino acids and can be found in the cytoplasm and cell nucleus [[18]. Several proteins have been identified that interact with the APC protein. These include: DLG protein, microtubule protein, GSKβ-3, β-catenin, γ-catenin, p34, Tid56, Axin protein. Interactions with many proteins indicate that the APC protein participates in the regulation of many cell processes including: cell division, migration, adhesion and cell fate determination [1]. A number of functional domains have been identified in the APC protein. The base domain encompasses amino acids 2200–2400 [1]. The N-terminal end of the protein, between amino acids 1 – 171, is involved in oligomerisation. There are two β-catenin binding sites in the APC protein – in the fragment comprising three 15-nucleotide repeats between amino acids 1020 and 1169 and in the region of 20-amino acid repeats between amino acids 1324 and 2075. Binding with microtubules, which occurs with increased gene expression, takes place with the assistance of the fragment encompassing amino acids 2130–2483. Amino acids 2450–2843 bind with EB1 protein, while amino acids 2771–2843 bind with DLG protein [1]. Attempts to identify the region associated with apoptosis have met with little success, although it has been reported that the expression of the appropriate APC protein in an intestinal neoplastic cell line leads to the occurrence of this phenomenon. A product of the *APC* gene of 300 kDa mass participates in the contact inhibition of cell growth in mucous cells of the large intestine.

**Figure 1:**
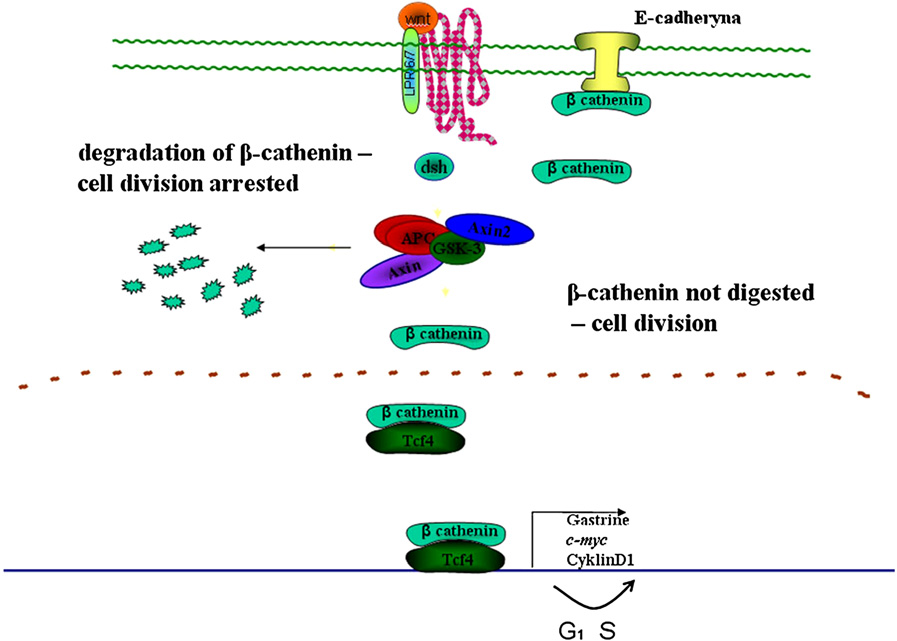
Role of the APC protein in cell cycle regulation.

Moreover, APC protein also interacts with γ-catenin and β-catenin. Both proteins bind with a cell adhesion protein – E-cadherin. Fearon (1997) proposed a model in which the APC protein participates in signal transduction and via β-catenin degradation affecting the activity of TCF4 (*T-cell transcription factor 4*) transcription factor (Figure 1) [20]. The protein that regulates the formation of the APC protein and β-catenin complex is protein kinase ZW3/GSK3β. Phosphorylation of the APC protein activates β-catenin binding. The activity of GSK3β kinase is regulated by DSH protein, which interacts with the protein products of *WNT1*. APC protein bound with kinase ZW3/GSK3β is capable of inhibiting the transcription induced by β-catenin. In the case of the loss of the *APC* gene product the transcription factor TCF4 (*TCF7L2*) becomes constitutively activated. Cells are stimulated to proliferate following activation of *c-MYC* by TCF4 [22]. The product of *c-MYC* resides in the cell nucleus and is capable of binding with DNA, activating a series of growth- associated genes that include ornithine decarboxylase (*ODC1*) and *CDC25A.* In addition, c-MYC inhibits *GAS1*. It was further shown that the activated β-catenin-TCF4 complex induces TCF1 expression [23]. In the large intestinal mucous cells *APC* serves as a negative regulator of the cell cycle via the regulation of β-catenin, which is normally activated by a proliferation signal derived from the transmembrane protein E-cadherin. When *APC* is lost the balance between cell division and apoptosis in the tissue becomes disturbed which results in uncontrolled cellular proliferation.

### Mutation of the *APC* gene

The development of molecular studies was triggered in 1991 by the discovery of the relationship between *APC* mutations and FAP. Over the past 11 years, the development of various techniques followed by increased numbers of publications has occurred in a growing number of countries and ethnic groups.

Investigations performed over several years using such techniques as single-stranded conformational polymorphism (SSCP) analysis, heteroduplex (HD) analysis or denaturing gradient gel electrophoresis (DGGE) method and more latterly DNA sequencing allowed the establishment of mutation databases. At the present time, the best *APC* gene mutation database resides at the Institute of Medical Genetics in Cardiff (http://www.hgmd.cf.ac.uk/ac/index.php) as it provides a comprehensive list of mutations for the majority of reported genes [24]. At present, information exists for 858 different *APC* mutations have been reported to the HGMD database. Small deletions constitute the highest proportion of *APC* mutations. A total of 356 mutations have been described and, in the majority, they lead to a change in the reading frame and the creation of a premature termination codon. Much rarer are large deletions (54), splice site mutations (49), small deletions combined with insertions (17), large insertions (7), complex rearrangements (6) and 3 mutations were observed in regulatory sequences. 235 mutations were represented as substitutions leading to changes in amino acids or to the development of the stop codon at the mutation site. The total of 131 small insertions have been described so far. In the case of substitutions, which constitute 30% of all mutations, the stop codon arises at the mutation site, whereas in the case of deletions or insertions, which constitute 68% of mutations, the STOP codon occurs downstream of the mutation as a result of a shift in the reading frame. There is a region in the gene characterised by an increased frequency of mutations called the MCR (*mutation cluster region*) which encompasses codons 1055–1309 and 23% of all germline mutations occur in this region. In addition, among germline mutations, an increased frequency of the following three mutations has been observed: deletions of 5 bp in codone 1309 (10%), deletions of 5 bp in codone 1061 and deletions of 4 bp in codone 1064 [18]. The majority of mutations can be found in the 5’ region of exon 15 of the *APC* gene. A characteristic FAP attribute is loss of heterozygosity (LOH) in the *APC* in adenomas developing as a result of somatic mutation in the second allele. The frequency distribution of these mutations differs from germline mutations. Up to 60% of somatic mutations are situated in the fragment, which encompasses 8% of the gene between codons 1286 and 1513. Somatic mutations occur in two hot spots in codon 1309 and codon 1450. Mutations in *APC* also occur in cases of large intestinal tumours unrelated with polyposis. In “sporadic” colorectal cancer (CRC) these neoplasms occur as a result of a mutation in one of the *APC* alleles followed by a loss of heterozygosity as a result of somatic mutations. In the case of the Lynch syndrome (HNPCC), the process is accelerated by inherited mutations in DNA repair genes [25]. According to the latest investigations, it is not necessary for LOH to occur in *APC* in order for the initiation of colorectal cancer to take place. In half of the sporadic cases of CRC in which no changes in *APC* occur, tumour development is associated with heterozygote mutations in the β-catenin gene (*CTNNB1*) [26]. These mutations result in the switching off of the APC regulatory function leading to β-catenin accumulation and, hence, expression of the *MYC* gene and development of CRC.

Attempts have been made to determine whether a genotype phenotype correlation exists that can be associated with the clinical course of the disease. It has been reported that the occurrence of the stop codon upstream of codon 157 is associated with a decreased number of polyps and a milder disease course. A typical disease course associated with hundreds to thousands of adenomas can be expected when the stop signal occurs between codons 169 and 1600. Mutations found between codons 1403 and 1587 lead to the diversification of symptoms that include osteomas, desmoid tumours and CHRPEs. The occurrence of desmoid tumours is also associated with mutations in the region between codons 1445 and 1578. Mutations at the 3’ end of *APC* result in a differing picture of disease both with regard to polyp numbers and the occurrence of extra-colonic symptoms.

### Progression of morphological and genetic changes in familial adenomatous polyposis

The inheritance of the mutated allele of the *APC* gene does not result in a clinical picture of the disease. The appearance of clinical symptoms is associated with the sequence of further events in the cell. Both in the case of FAP and HNPCC, cells are characterised by a very high risk of loss of the wild type *APC* allele. Gene deletions in tumours are observed to occur in regions associated with proliferation located on 5q (*APC, MCC*), 17p (*TP53*) and 18q (*SMAD4*) chromosomes, while point mutations are often found within the *K-RAS* prooncogene. In the case of FAP, the risk of loss of heterozygosity is very high because one of the alleles is inherited in a mutated form. The loss of a series of genes associate with cell-cell recognition, DNA damage response and cell proliferation must occur prior to tumour development. It is not the order that is important, rather the total number of events. Of particular interest is the necessity of APC loss prior to carcinoma development. It has been reported that a series of genes can be lost (P53, MCC, k-RAS and DCC) but without APC tumour development does not occur [6]. Once APC is lost, tumour development is inevitable.

### Structure and location of the *MUTYH* gene

The *MUTYH* gene (MutY, e. coli, Homolog) is a homologue of a bacterial *mutY* gene involved in the repair of oxidative DNA damage. Alternatively, the *MUTYH* gene is frequently referred to as *hMYH* or *MYH* but the above names are considered as inappropriate due to their being used to describe myosin genes. In man, the *MUTYH* gene was first described in 1996. It encompasses 7100 bp on chromosome 1 in the region of p34.3-p32.1 and consists of 16 exons. None of the 15 introns of the *MYH* gene exceed 200 bp. The gene encodes a 535-amino acid protein, which is 41% identical with the *mutY* protein found in *E. coli* not. Most frequently, two substitutions Y165C and G382D occur which are observed in 80% of patients with mutations of both gene alleles [5,27,28].

### Phenotype mutation in the APC gene (Phenotype associated with mutations in the APC gene)

#### Familial adenomatous polyposis (FAP) (MIM 175100)

Familial polyposis of the large intestine (FAP) is a predisposition inherited in an autosomal dominant manner for the occurrence of numerous polyps in the large intestine. Symptoms in the form of numerous (hundreds or thousands) polyps in the mucous of the large intestine appear towards the end of the second decade of life. Average age of polyp incidence is 15 years of age [25]. The polyps are characterized by large potential for the development towards malignant tumour. Malignancy can occur from late childhood onwards. The first symptoms of FAP are diarrhea and blood in the stool. Weight loss and weaknesses occur after the development of advanced tumour. The incidence of the FAP disorder is one per 10000 newborns. There are high levels of heterogeneity with regard to the number and timing of the occurrence of polyps. The typical disease course is characterised by the occurrence of over 100 polyps, which appear most frequently in the second decade of life and can co-occur with lymphoid polyps [29]. The most frequent polyps are tubular adenomas with diameters reaching up to several centimetres. Tubular-villous and villous adenomas are also quite frequent [30]. Moreover, intestinal polyps can also occur as flat tubular polyps [31]. One of the typical features of polyposis is a severe phenotype in which the number of polyps exceed one thousand [32]. Severe FAP is characterised by an earlier age of tumour occurrence, on average 34 years of age, and a more frequent occurrence of extra-colonic disease.

Extra colonic features of FAP vary considerably and can include:

● Retinal pigment discolouration of the eye fundus (*congenital hypertrophy of the retinal pigment epithelium* - CHRPE) is has been tentatively associated with the location of the mutation in the *APC* gene. CHRPE has been reported to occur in more than half of the carriers of the mutation in the *APC* gene [33], however, this remains to be confirmed. Retinal hyperpigmentation has not been shown to occur if the product of the mutated gene is smaller than 50 kDa and exon 9 is considered as the boundary between mutations causing or not causing discolouration. In the 3’ region of the *APC* gene, codon 1387 is the boundary of the occurrence of hyperpigmentation [34]. The length of the product affects the discolouration picture. Gene protein products less than 1014 amino acids long present a picture of a small round pigmented spot or large round pigmented spot. Protein products encompassing at least 1014 amino acids are associated with an increased incidence of the remaining two types of retinal changes in fundus, i.e. oval pigmented with a surrounding halo and large round de-pigmented spot [35]. Codon 1014 is the binding site of two cytoplasmic proteins: α and β-catenin, which are important for protein active in the adhesion of E-cadherin cells. Products longer than 1014 amino acids can bind with these proteins, which may affect the change of phenotype discolouration [36]. Retinal hyperpigmentation is used as a carrier state marker of the mutated allele, although the development of molecular biology techniques as well as incomplete penetrance has reduced its significance.

● Changes in dentition involving, most frequently, the appearance of supernummery teeth as well as osteomas and changes in tooth structure,

● Polyps in upper segments of the gastrointestinal tract. Duodenal and gastric polyps can occur in the form of polyps of the stomach fundus or adenomas [37]. Stomach polyps occur in approximately 50% of FAP patients and do not have a high potential to form neoplasms. Adenomas observed in 6% of cases form neoplasms even less frequently than polyps of the stomach fundus [38]. Duodenal polyps are diagnosed in 33% to 44% of FAP patients [39-41], although a group of probands was described where the frequency of duodenal polyps reached 80% [41]. Polyps are usually situated in the descending part of the duodenum. In some of patients, larger polyps are observed in the vicinity of the larger papilla of the duodenum.

● Desmoid tumours are observed in about 10% FAP patients and its appearance usually follows a surgical operation [42]. In comparison with general population, desmoid tumours are observed at significantly increased rates in FAP patients. Moreover, gender is also found to modify the occurrence frequency; the disease occurs less frequently in men with FAP than in women at a ratio of 1: 3 [42,43].

● Thyroid gland tumours are observed in FAP with more than 94% of cases being diagnosed in women [44-46].

● Central nervous system neoplasms occur with low frequency of about 1% and they are usually gliomas. The occurrence of these tumours together with FAP symptoms in the intestine is described as Turcot’s syndrome (MIM#276300) [47-49]. Brain cancer in FAP is associated more with the occurrence of the locus modifying the disease course than with a specific spectrum of mutations in the *APC* gene. This is exemplified by the fact that in cases of sporadic brain gliomas or medulloblastomas no mutations in the *APC* gene have been reported [50,51].

● Hepatoblastomas are rare neoplasms occurring in children. Increased risk of occurrence of these cancers in *APC* mutation carriers were observed but the incidence did not exceed 1% [32,52,53].

### Attenuated form of familial adenomatous polyposis coli (AAPC)

The mild form of the large intestine familial polyposis (*attenuated adenomatous polyposis coli* – AAPC) is characterised by the occurrence of a small number of polyps, from several to one hundred. From among parenteral symptoms, the occurrence of the stomach fundus polyps is rare [34]. This form of familial intestinal polyposis is connected with the occurrence of mutation at the 5’ end of the *APC* gene. It is assumed that codon 159 is the boundary between AAPC and FAP causing mutations, although it is difficult to establish this boundary unequivocally because this form of the disease occurred also in cases when the mutation led to the formation of the Stop codon in exon 9 of the *APC* gene [34].

### Phenotype of the *MUTYH* gene mutation

Recessive polyposis (MIM 608456) / MAP (MUTYH associated polyposis).

Recessive polyposis is an autosomal recessive predisposition for the occurrence of numerous polyps in the large intestine whose numbers are smaller than in the case of FAP and do not exceed 100 [54]. Cancer risk incidence increase in mutation carries of both *MUTYH* gene alleles and it is 93 times higher in comparison with general population; intestinal neoplasm appears nearly always before the 60^th^ year of life [55]. Moreover, it was observed that mutations of both *MUTYH* gene alleles increase the risk of endometrial tumours which, at a small number of polyps or their absence, may make the phenotype resemble Hereditary Nonpolyposis Colorectal Carcinoma (HNPCC) [55]. Differences in the extent of pathogenic symptoms are also observed in the relatives of carriers of the same mutations [55].

### Turcot syndrome (MIM 276300)

The Turcot syndrome comprises the co-occurrence of neoplasms of the gastrointestinal tract and malignant carcinoma of the central nervous system. In 1991, two forms of the disease were identified: one inherited in an autosomal dominant manner with the occurrence of medulloblastoma at young age and intestinal FAP symptoms and the other inherited autosomally in a recessive manner with the occurrence of glioblastoma and HNPCC attributed to elderly patients in the large intestine [56]. It was observed in Turcot syndrome that in the case of patients with a mutation in the *APC* gene, in 79%, a medulloblastoma appeared, whereas in patients with the intestinal traits indicating mutations in the DNA repair genes – a glioma developed. Mutations identified in the *APC* gene exhibit heterogenity. No specific region in the *APC* gene was identified associated with the occurrence of the Turcot syndrome.

### FAP molecular diagnosis

DNA isolated from peripheral blood cells is used for the molecular analysis of *APC* gene. The search for mutations employs screening methods that include heteroduplex analysis (HA), single-stranded conformation polymorphism (SSCP) and high resolution melting (HRM). HRM is one of the more novel techniques employed in screening investigations which allows identification of small sequence changes within the examined fragments [57]. The mode of action of the method is based on monitoring the behaviour of DNA fragments, amplified earlier, during the denaturation process. The analysis is enabled by the presence in the reaction mixture of a fluorescent dye which intercalates only the double-stranded DNA sending a strong fluorescent signal making it possible to observe the melting process, i.e. conversion of dsDNA into ssDNA in the course of denaturation. The comparison of melting profiles of individual fragments with one another makes it possible to select those that indicate differences in the course of denaturation which, at the same time, reflect changes in the sequence. HRM is a screening technique and fails to provide accurate information about changes in the sequence and, therefore, all atypical melting profiles require confirmation by sequencing. Nevertheless, high sensitivity of the method, according to some researchers, reaching even 100%, allows considerable reduction of sequencing numbers and, consequently, of the cost of analyses, at the same time maintaining and sometimes even increasing the identification effectiveness of mutations in comparison with earlier screening methods.

An important issue is the detection of large rearrangements in the *APC* gene. Searching for large changes concerning particular exons or even entire genes was greatly simplified when in 2002, Schouten described Multiplex Ligation-dependent Probe Amplification (MLPA) the quantitative analysis of up to 40 DNA fragments amplified with identical primers in the same reaction tube [58]. Since then, there has been a rapid development of this technique and the method is currently used in research on predispositions to hereditary diseases, characteristic of cancer, methylation analysis, RNA analysis and pharmacogenetics.

The method is based on hybridization of probes halves to selected fragments of genomic DNA, their ligation and amplification. This allows the quantitative evaluation of PCR products. Hybridization of the probes to a specific DNA fragment opens up possibilities for the study of known mutations that occur with increased frequency in cases of certain diseases.

The MLPA kit P043 for the *APC* gene in addition to probes for testing particular exons contains two probes for the detection of mutations 3183-3187delACAAA and 3927-3931delAAAGA. These two mutations are, in some populations even up to several percent of all detected mutations in FAP patients. In our group of patients with FAP, these two mutations are present almost in 15% of patients.

The next-generation sequencing opens new possibilities in the search for mutations in the *APC* gene. This method with target enrichment allows a detailed examination of the gene sequence. The advantage this method is ability to detect of small mutations as well as those involving several exons. Such tests have been developed. One of them is ColoSeq, where the target enrichment and mass sequencing allow test at the same time all of the genes of suitability for FAP and HNPCC [59].

**Figure 2:**
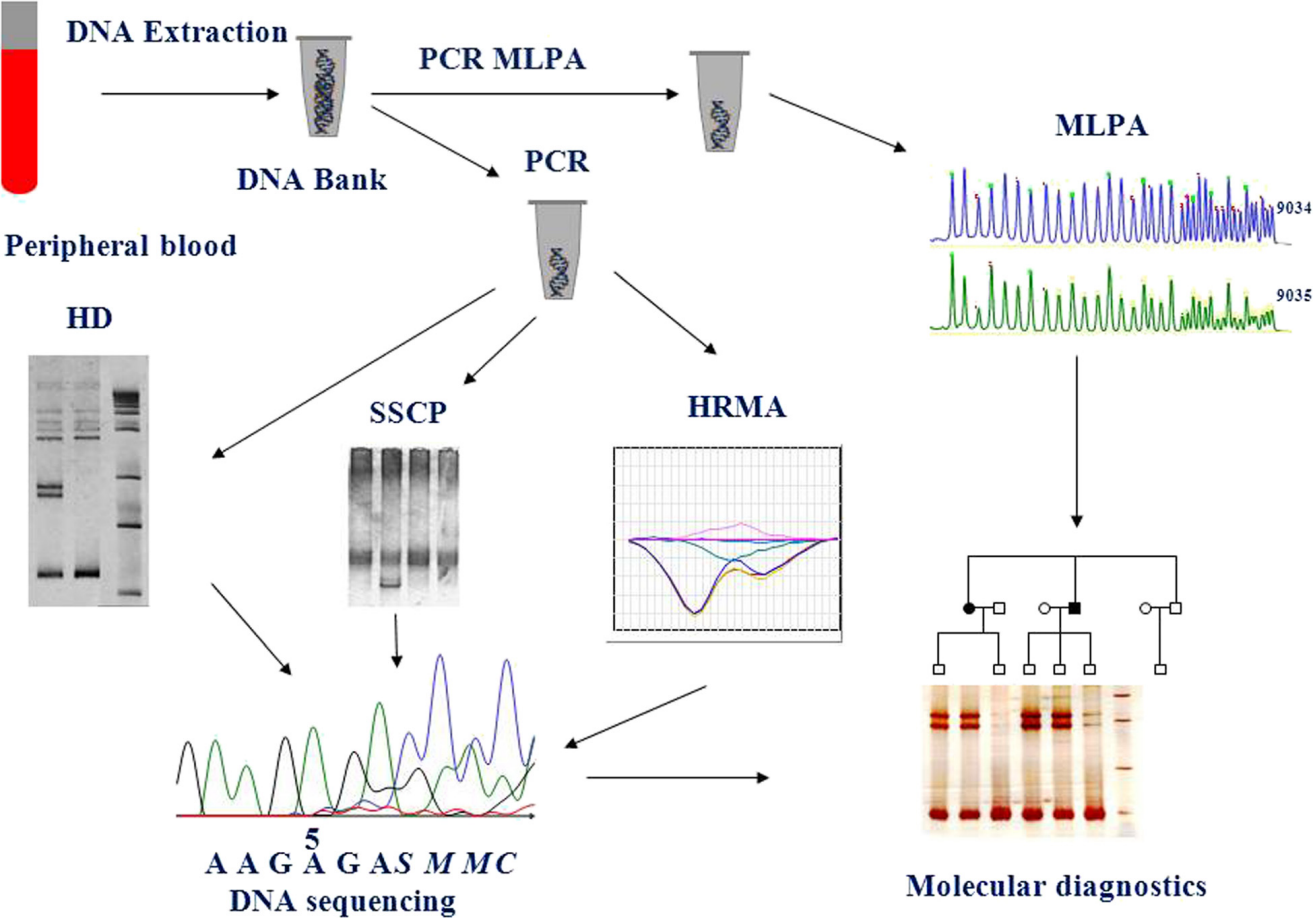
Molecular diagnostic of FAP.

After the identification of mutation in the *APC* gene, an analysis of inheritance of the mutated allele of the *APC* gene is performed in all persons of the probands’ family (Figure 2). In the situation where no mutations are identified, indirect analysis can be applied that examine the co-inheritance of conjugated markers [60]. Markers located close to the examined gene are applied to investigate inheritance of the mutated allele. It is recommended to apply several gene flanking markers to avoid mistakes resulting from crossing-over between the marker and the gene. Familial adenomatous polyposis has been investigated in our country for over 15 years now at the IHG PAS in Poznan. The established DNA Bank of Polish patients suffering from familial polyposis contains material from 600 families. Research results have been presented in specialised journals [45,61]. Approximately 50% of FAP families without an identified *APC* mutation pose a diagnostic problem. Some reports indicate new research directions in this field. Dutch scientists succeeded in identifying miRNAs, which regulate *APC* gene expression. Target sequences for miRNA as well as the location of 3 encoding genes were determined. The gene coding miR-135a-1 is located in the first intron of the stabilin 1 gene *STAB1* (3q21), whereas the gene coding miR-135a-2 is found in intron 5 of the *RMST* gene coding the transcript associated with rabdomyosarcoma (12q23) [62]. The miR-135b coding gene is located on chromosome 1q32.1 in intron 1 of the *LEMD1* gene. miRNA precursors are made up of 93, 102 and 99 nucleotide sequences and can be found on chromosome 3 - miR135a-1, on chromosome 12 miR-135a-2 and chromosome 1 miR-135b, respectively. It has been shown experimentally that in *in vitro* miRNA system – miR135a and miR135b interact with 3’UTR transcript of the *APC* gene and affect the level of mRNA and, in this way, regulate APC expression and consequently the *Wnt* proliferation control pathway. Furthermore, it was observed that increased quantities of miR-135a and miR-135b lead to the reduction in the amount of mRNA of the *APC* gene in neoplastic tumours of the intestine [63]. Examination of these sequences will increase our knowledge about the genetic background of this disease and improve its diagnosis. The most recent reports indicate the potential involvement of changes outside the coding sequence in FAP conditioning because their impact on the correctness of expression may be important.

### FAP prophylaxis

At the present time patients at risk of disease are screened by sigmoidoscopy every 12 months beginning at puberty. This makes it possible to discover symptoms long before the development of an invasive neoplasm. Once polyps appear, the only effective measure to prevent the occurrence of the malignant disease is to remove surgically the entire colon and rectum. Prophylactic colectomy performed at an appropriate age prolongs the life expectancy of FAP patients, on average, from 45 to over 60 years of age.

During surgery, the entire large intestine is removed and a *J pouch* is formed using the small intestine. The mucous membrane of the small intestine exhibits considerable plasticity and it becomes morphologically similar to the mucous membrane of the large intestine. Surgery is currently the only possibility to improve the life expectancy of patients, although very effective, it causes serious morbidity, especially when performed at advanced stages of disease when the anus must also be removed. There is much interest in alternative approaches where there is an ongoing assessment of the impact of food composition and non-steroid anti-inflammatory drugs shown to reduce the numbers of intestinal adenomas. Interestingly, resistant starch consumption and the incidence of large intestinal tumours demonstrate an inverse correlation and animal studies have shown some promise. A number of mouse models have been developed, including a functional/knock-out gene *APC*^Δ^*716*[64]. These mice are heterozygotes for the *APC* mutation at codon 716 which results in a typical disease course in terms of polyp appearance occurring at about the third week of life in the mouse. Homozygote mutation carriers are embryologically lethal. Diets containing high levels of starch and low in fat did not affect the frequency of disease but did reduce by 64% polyp numbers as well as their size in this mouse model. Numbers of polyps are also reduced by the administration of non-steroid anti-inflammatory drugs. The application of Aspirin, Piroxicam and Sulindac reduced the number of polyps in the large intestine of experimental mice with mutated *APC* from 40% to 60% [63].

Attempts are also being undertaken to employ gene therapy for FAP treatment. The fact that the illness is preconditioned by a mutation in one gene facilitates significantly attempts to repair the error. Using liposomes as vectors, a complete functional *APC* gene was introduced into a colorectal cancer cell (SW480) line derived from the large intestine with a mutated *APC* gene. The transfer of *APC* led to the expression of the correct APC in SW480 cells after 72 hours and it was observed for a period of one week at a level guaranteeing a biological effect.

Neoplastic diseases of the large intestine are well recognised but it remains necessary to understand all factors influencing the initiation and development of tumours as well as possible interactions between these factors. Studies of mutations occurring in the course of the development of neoplastic diseases will make it possible to select the most affective prophylactic method or minimisation of pathologic consequences.

## Competing interests

The authors declare that they have no competing interests.

## Authors’ contributions

All authors contributed to the literature search and manuscript preparation. All authors read and approved the final manuscript.
